# NIR‐Responsive Gold Nanorod Capsules for Efficient Cargo Encapsulation and Release

**DOI:** 10.1002/smll.75754

**Published:** 2026-09-23

**Authors:** Dongyu Zhang, Takehiro Yachi, Daisuke Unabara, Tasuku Hamaguchi, Koji Yonekura, Kiyoshi Kanie, Kuniharu Ijiro, Hideyuki Mitomo

**Affiliations:** ^1^ Graduate School of Life Science Hokkaido University Sapporo Japan; ^2^ Institute of Multidisciplinary Research for Advanced Materials Tohoku University Sendai Japan; ^3^ Biostructural Mechanism Laboratory Hyogo Japan; ^4^ International Center for Synchrotron Radiation Innovation Smart Tohoku University Sendai Japan; ^5^ Research Institute for Electronic Science Hokkaido University Sapporo Japan

**Keywords:** cargo encapsulation, drug delivery systems, gold nanorods, near‐infrared‐triggered release, plasmonic nanocapsules, self‐assembly

## Abstract

Gold nanoparticle‐based vesicular nanostructures are attractive drug delivery platforms because of their tunable plasmonic properties; however, simultaneously achieving efficient cargo encapsulation, robust aqueous stability, and controllable near‐infrared (NIR)‐triggered release remains challenging. Herein, water‐dispersible and NIR‐responsive gold nanorod capsules (Au NrCs) are fabricated from mini‐gold nanorods (mini‐Au NRs) and small amphiphilic hexaethylene glycol (EG6) ligands via a solute‐induced phase separation strategy. Owing to their compact dimensions and tunable longitudinal localized surface plasmon resonance, the mini‐Au NRs enable assembly within sub‐200 nm nanocapsules while retaining NIR responsiveness. During capsule formation, nanoparticles and DNA cargoes are simultaneously encapsulated and concentrated within the capsules through liquid‐liquid phase separation, achieving approximately 90% DNA encapsulation efficiency and more than 1000‐fold enrichment. Furthermore, amino‐terminated EG6 ligands enable post‐assembly crosslinking of the Au NrCs with diepoxy‐polyethylene glycol, improving aqueous stability and dispersibility. The resulting Au NrCs exhibit efficient cargo release (>95% within a few minutes) under low‐power 810 nm NIR LED irradiation through capsule disassembly, highlighting the potential of plasmonic nanocapsules for externally controlled delivery under relatively mild NIR LED irradiation conditions. These results establish a plasmonic nanocarrier platform integrating concentration‐assisted cargo encapsulation, enhanced aqueous stability, and controllable NIR‐triggered release, providing a basis for future drug delivery applications.

## Graphical abstract

Mini‐gold nanorods modified with oligo(ethylene glycol) ligands self‐assemble into plasmonic nanocapsules through a solute‐induced phase separation process. Subsequent crosslinking with bifunctional polyethylene glycol improves aqueous stability while preserving efficient cargo encapsulation and near‐infrared (NIR)‐triggered release. The resulting nanocapsules achieve approximately 90% DNA encapsulation together with more than 1000‐fold enrichment and rapid cargo release under low‐power NIR irradiation.

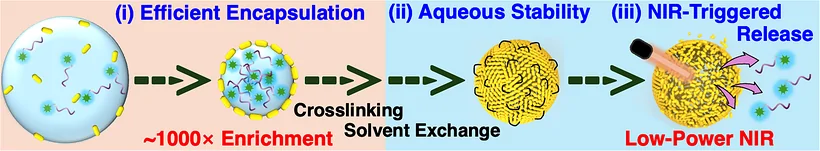

## 1 Introduction

Developing nanocarriers that simultaneously achieve controlled nanoscale dimensions, efficient cargo loading, and externally controllable release remains an important challenge in advanced drug delivery systems (DDS) [1, 2, 3, 4, 5, 6], particularly for the delivery of nucleic acid therapeutics [7, 8, 9]. Unlike many small‐molecule drugs, nucleic acids are highly hydrophilic, polyanionic macromolecules that are inherently difficult to incorporate into nanocarriers with high loading capacity. Consequently, current loading strategies often rely on intermolecular interactions, such as electrostatic complexation with cationic carriers or adsorption onto nanomaterial surfaces, to facilitate nucleic acid incorporation [10, 11]. Although these approaches can promote cargo loading through localized enrichment, they are generally associated with limited loading capacity and may complicate efficient cargo release because of the strong interactions between the carrier and cargo. Therefore, alternative strategies that enable efficient physical enrichment and encapsulation of nucleic acid therapeutics without relying on strong carrier–cargo interactions are highly desirable.

Vesicular nanostructures are attractive DDS platforms because their hollow 3D interiors enable physical encapsulation and protection of cargoes while offering the potential for larger loading capacities than surface‐based loading strategies [12, 13]. Capsule‐based carriers ranging from micrometer to nanometer scales have been extensively investigated for cargo encapsulation and controlled release [14]. However, sub‐200 nm nanocapsules are generally considered desirable for systemic drug delivery because they provide a favorable balance between prolonged blood circulation and reduced nonspecific clearance, while their fabrication with well‐controlled dimensions and efficient cargo loading remains challenging. Conventional organic vesicular systems, such as liposomes [15, 16, 17, 18], have demonstrated excellent biocompatibility and broad applicability in drug delivery [19, 20, 21]. However, many of these systems primarily rely on passive responses to biological environments [22], such as pH [23, 24, 25] or redox conditions [22, 26, 27], and their responsiveness to externally applied stimuli is often limited [28, 29]. Therefore, externally triggered release systems that enable on‐demand control over cargo release have attracted increasing interest [30]. In particular, inorganic nanoparticle‐based vesicular systems capable of externally triggered release, such as those responsive to light or magnetic fields [31, 32, 33, 34], have attracted increasing interest because they enable remote and controllable release behavior [35, 36, 37, 38].

Among inorganic nanomaterials, gold nanoparticles (Au NPs) are particularly attractive building blocks for light‐responsive vesicular DDS platforms because of their high biocompatibility, tunable optical properties, and efficient photothermal responsiveness [32, 39]. In particular, amphiphilic polymer‐directed self‐assembly has enabled the construction of various hollow plasmonic vesicular structures based on Au nanoparticles [40, 41, 42, 43]. Furthermore, Au nanorod (Au NR)‐based assemblies capable of near‐infrared (NIR)‐responsive cargo release have attracted increasing attention owing to the deeper tissue penetration and lower phototoxicity of NIR light [33, 43, 44, 45]. However, cargo loading in these systems generally relies on conventional loading approaches, in which cargo molecules are incorporated into preformed or forming nanocarriers without active concentration during capsule formation [44, 45, 46, 47]. Such approaches do not actively concentrate hydrophilic cargoes during capsule formation, making it difficult to achieve high loading capacities for highly hydrophilic biomacromolecules such as nucleic acids. Consequently, strategies capable of enriching hydrophilic cargoes during nanocarrier formation, rather than after carrier fabrication, are highly attractive for achieving efficient loading while increasing the amount of cargo encapsulated within hollow nanocarriers. In addition, most reported NIR‐responsive Au NR delivery systems rely on high‐power laser irradiation and are primarily designed for photothermal therapy [42, 43, 47, 48]. Therefore, plasmonic vesicular nanocarriers that simultaneously achieve efficient cargo encapsulation and controllable release under relatively mild NIR irradiation conditions remain highly desirable for biomedical delivery applications.

While most plasmonic nanocapsules have been constructed using amphiphilic polymers [40, 41, 42, 43], we have developed an alternative strategy based on small‐molecule amphiphilic ligands, enabling the facile formation of densely assembled plasmonic nanocapsules through a simple molecular design [39, 49]. Building on this strategy, we recently developed a solute‐induced phase separation approach using biocompatible oligo(ethylene glycol)‐based ligands together with kosmotropic ions, enabling the spontaneous formation of ∼200 nm Au nanocapsules in water‐miscible organic solvents [50]. In this previously reported spherical nanoparticle system, the internal aqueous phase was simultaneously concentrated during capsule formation, enabling highly efficient encapsulation and more than thousand‐fold enrichment of hydrophilic cargoes. This concentration‐assisted encapsulation behavior provides a promising strategy for increasing the loading of water‐soluble therapeutics, particularly nucleic acids, within hollow nanocarriers. However, these nanoparticle capsules relied on solvent asymmetry and nanoparticle amphiphilicity to maintain their structural integrity, resulting in capsule disassembly upon transfer into aqueous environments [47, 50]. In addition, because the capsules were constructed from spherical Au nanoparticles, efficient NIR‐responsive release under mild irradiation conditions remained difficult to achieve [39, 47, 50, 51]. Therefore, extending this concentration‐assisted encapsulation strategy to water‐dispersible, NIR‐responsive plasmonic nanocapsules remained an important challenge.

To address these challenges, in this study, we fabricated water‐dispersible, NIR‐responsive gold nanorod capsules (Au NrCs) based on mini‐Au nanorods (mini‐Au NRs) and amphiphilic hexaethylene glycol (EG6) ligands using our previously established solute‐induced phase separation strategy (Scheme 1). Owing to their compact dimensions and tunable longitudinal localized surface plasmon resonance (L‐LSPR), mini‐Au NRs enable assembly within sub‐200 nm nanocapsules while retaining efficient NIR photoresponsiveness. During self‐assembly, model cargoes, including nanoparticles and DNA molecules, were simultaneously encapsulated and enriched within the capsule interior through solute‐induced phase separation. Furthermore, amino‐terminated EG6 ligands were introduced to enable post‐assembly crosslinking with diepoxy‐polyethylene glycol (diepoxy‐PEG), thereby improving the aqueous stability and dispersibility of the Au NrCs. The resulting plasmonic nanocapsules enabled efficient cargo release under low‐power NIR (810 nm) LED irradiation through capsule disassembly, demonstrating the potential of plasmonic nanocapsules for externally controlled delivery under relatively mild NIR LED irradiation conditions.

**SCHEME 1:**
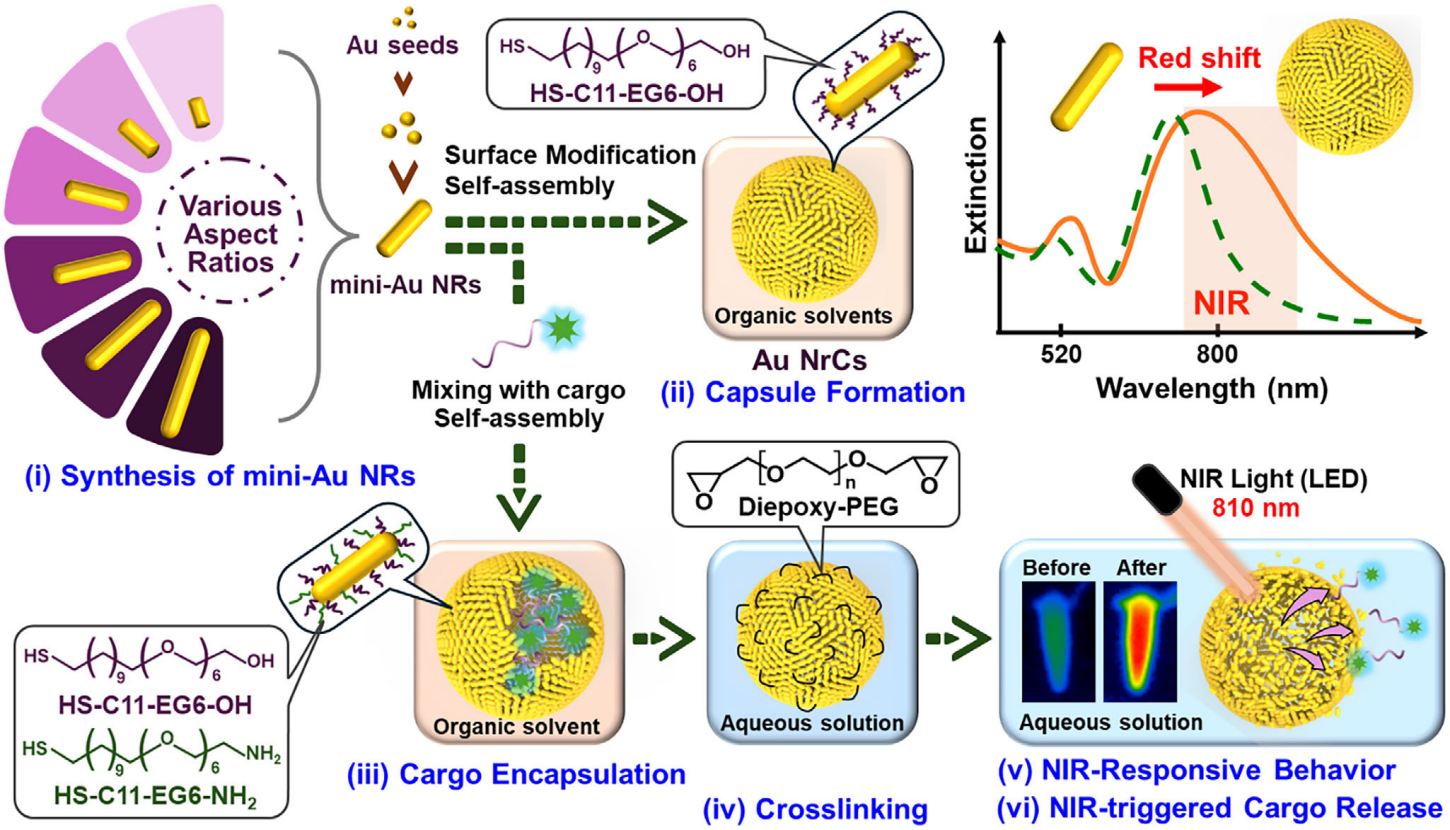
Schematic illustration of the fabrication and NIR‐responsive functionality of Au NrCs.

## 2 Results and Discussion

### 2.1 Synthesis of Mini‐Au NRs with Various Aspect Ratios

To enable tunable plasmonic responses of the nanoparticle capsules, Au NRs were selected as building blocks. Mini‐Au NRs with controlled sizes and aspect ratios (ARs) were employed to facilitate the formation of well‐defined hollow capsules, as larger particles tend to reduce the internal cavity volume and increase interparticle spacing, which can limit efficient cargo encapsulation. By adjusting the dimensions and ARs, the longitudinal localized surface plasmon resonance (L‐LSPR) could be systematically tuned from the visible to the NIR region. The synthesis was adapted from the method reported by González‐Rubio et al. with minor modifications and carried out in aqueous solution [52]. The procedure involved seed formation, secondary growth, and subsequent anisotropic growth to produce small‐sized Au NRs (Figure 1a). The Au seeds prepared by NaBH_4_ reduction exhibited no distinct extinction peak in the 400–600 nm region, consistent with their small size (Figure S1). After secondary growth, an LSPR peak at approximately 513 nm emerged, indicating the formation of larger seeds. Mini‐Au NRs with different ARs were obtained by tuning the concentrations of AgNO_3_ and seed solution during anisotropic growth. Increasing the AgNO_3_ concentration promotes longitudinal growth while maintaining the rod diameter, resulting in higher ARs [53]. Because the presence of HCl in the growth solution leads AgCl precipitation at high AgNO_3_ concentrations, both the AgNO_3_ concentration and the seed amount were adjusted to maintain stable growth. As a result, mini‐Au NRs with well‐defined rod‐like morphologies were successfully synthesized, as confirmed by high‐angle annular dark‐field scanning transmission electrion microscopy (HAADF‐STEM) images (Figure 1c and Figure S2). Statistical analysis of the HAADF‐STEM images revealed lengths ranging from 9 to 28 nm and ARs from 1.5 to 5.0, with narrow size distributions for each sample (Figure S3). The corresponding extinction spectra (Figure 1b) shows L‐LSPR peaks spanning from the visible to the NIR region, with intervals of approximately 40 nm, demonstrating precise control over optical properties. These characteristics make the mini‐Au NRs suitable building blocks for subsequent interfacial self‐assembly.

**FIGURE 1:**
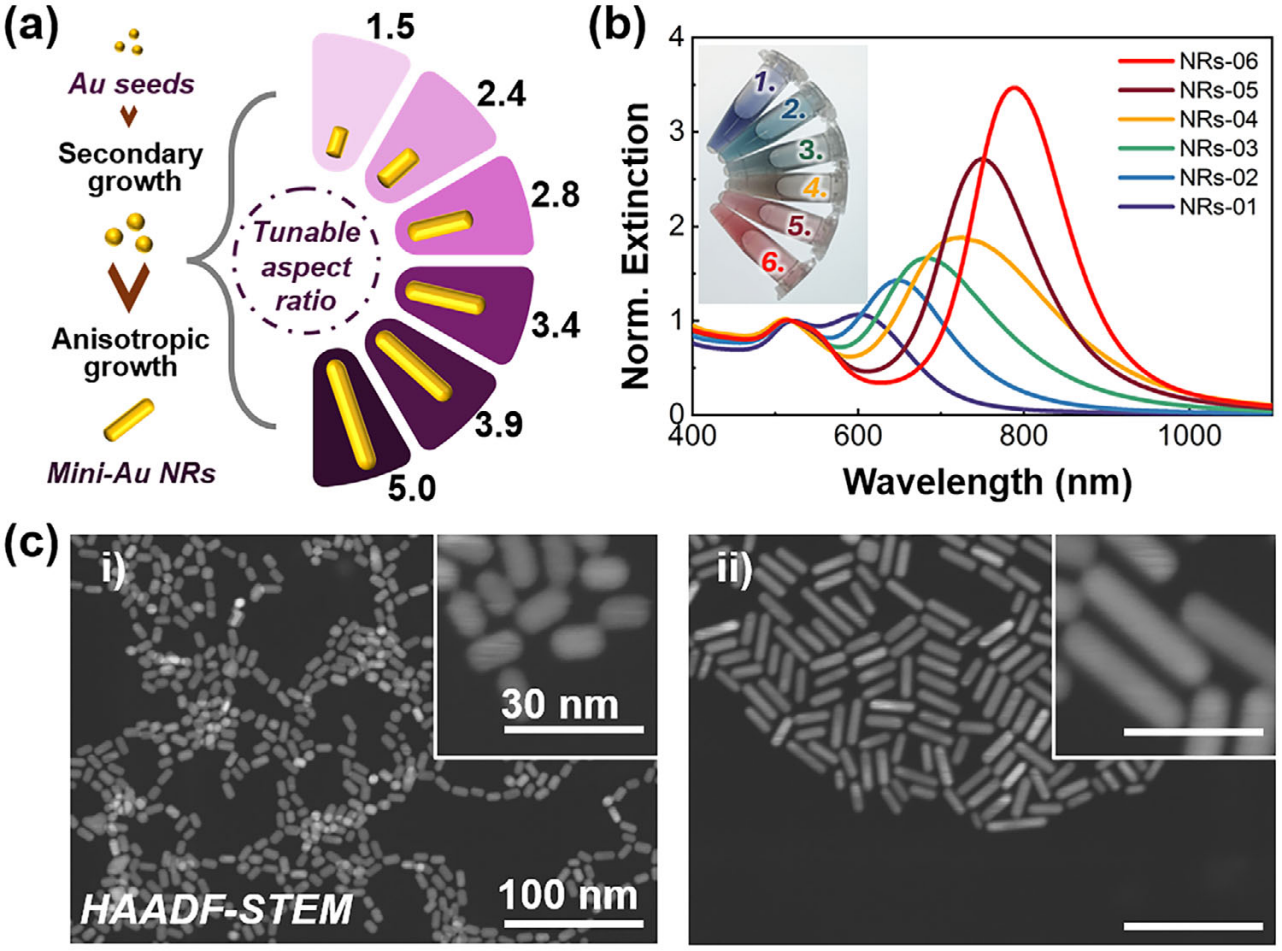
(a) Schematic illustration of the synthesis of mini‐Au NRs. (b) Extinction spectra of mini‐Au NRs with L‐LSPR peaks spaced at intervals of 40 nm (normalized at the T‐LSPR peak); inset shows the corresponding photograph. (c) HAADF‐STEM images of mini‐Au NRs: (i) sample 1 and (ii) sample 6 in Figure 1b. The scale bar in (ii) is identical to that in (i).

### 2.2 Capsule Formation via Self‐Assembly of Mini‐Au NRs

The formation mechanism of Au NrCs via the self‐assembly of mini‐Au NRs is illustrated in Figure 2a [50]. Briefly, cetyltrimethylammonium bromide (CTAB)‐capped mini‐Au NRs were subjected to ligand exchange with an amphiphilic ligand (EG6), followed by purification to remove excess ligands and surfactants, and subsequent addition of a kosmotropic salt (sodium citrate). Upon introduction into a miscible organic solvent (e.g., THF, dioxane), water from the aqueous phase gradually diffuses into the surrounding organic phase, while the solutes (nanorods and salts) are retained. This process, driven by the gradual extraction of water into the surrounding organic phase, leads to a progressive increase in local solute concentration within the shrinking aqueous domains, inducing phase separation and the formation of nanoscale droplets. Amphiphilic nanoparticles accumulate at the liquid–liquid interface and undergo interfacial self‐assembly, yielding hollow capsule structures.

**FIGURE 2:**
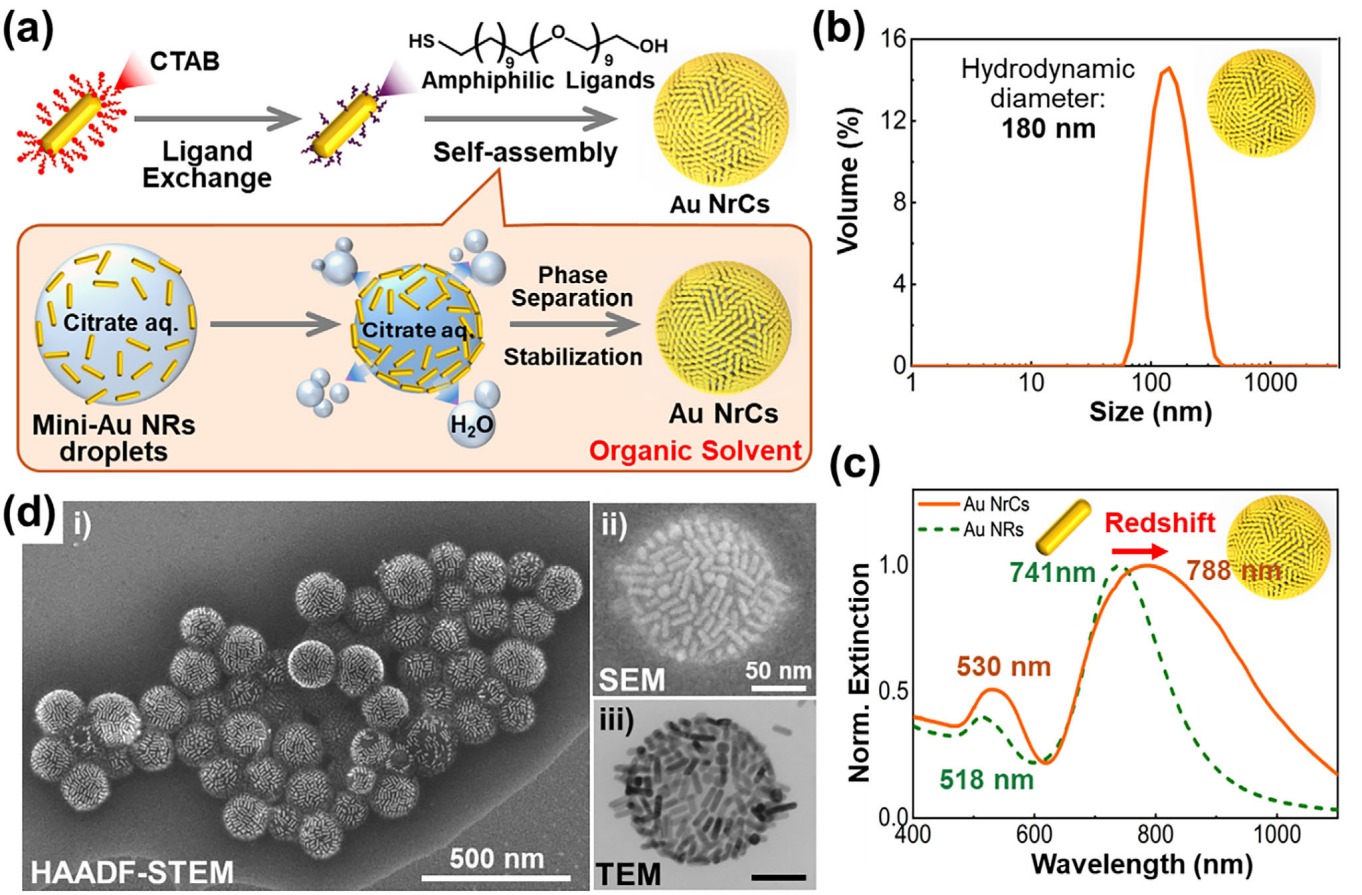
(a) Schematic illustration of the self‐assembly of mini‐Au NRs into Au NrCs. (b) DLS size distribution of Au NrCs in dioxane. (c) Extinction spectra of mini‐Au NRs before and after assembly (green dashed line: mini‐Au NRs; orange solid line: Au NrCs), normalized at the L‐LSPR peak. (d) Electron microscopy images of Au NrCs: (i) HAADF‐STEM image at low magnification; (ii) SEM image; (iii) TEM image. The scale bar in image (iii) is the same as that in image (ii).

To examine the formation of such assemblies in solution, dynamic light scattering (DLS) measurements were first performed. As shown in Figure 2b, the resulting structures exhibit an average hydrodynamic diameter of approximately 180 nm, indicating the formation of nanoscale assemblies in solution, consistent with capsule formation observed in previously reported systems using spherical nanoparticles [50]. The corresponding extinction spectra show that both the transverse (T‐LSPR) and longitudinal (L‐LSPR) bands undergo red shifts after assembly (Figure 2c), suggesting plasmonic coupling between adjacent nanorods due to reduced interparticle spacing. These observations indicate the formation of closely packed nanoparticle assemblies in solution. To directly visualize the assembled structures, electron microscopy analyses were conducted (Figure 2d). HAADF‐STEM image reveals well‐defined spherical structures with smooth boundaries, consistent with the formation of capsule‐like assemblies. The average diameter determined from HAADF‐STEM is 98.7 ± 19.0 nm, indicating a relatively uniform size distribution (Figures S4 and S5). The capsule sizes determined by both HAADF‐STEM and DLS are comparable to those reported for our previously developed nanoparticle capsules, supporting the formation of well‐defined sub‐200 nm Au NrCs [50]. SEM observations further indicate that the capsule surfaces are densely covered with nanorods. The contrast observed in TEM images suggests the presence of a hollow interior, consistent with the expected vesicular structure [39]. To exclude potential structural artifacts arising from drying during sample preparation, cryo‐TEM observations were performed on samples dispersed in organic solvent (Figure S6). The cryo‐TEM images confirm the presence of hollow spherical shells, consistent with the TEM observations, thereby supporting the formation of stable capsule structures in solution. Closer inspection by STEM reveals that mini‐Au NRs are densely and randomly packed on the capsule surface (Figure 2d), providing structural insight into the plasmonic coupling behavior observed in the extinction spectra. Such arrangements suggest the coexistence of multiple interparticle configurations, including side‐by‐side and tip‐to‐tip orientations. This structural heterogeneity is consistent with the optical behavior described above. In particular, side‐by‐side interactions are expected to induce blue shifts, whereas tip‐to‐tip interactions lead to red shifts; the experimentally observed slight overall red shift can therefore be interpreted as a combined effect of these competing coupling modes. The reproducibility of the fabrication process was further confirmed by three independent preparations, which exhibited nearly identical extinction spectra, DLS size distributions, polydispersity indices, and capsule morphologies (Figure S7), demonstrating the high reproducibility of the present fabrication strategy.

In this study, mini‐Au NRs with dimensions of 20.7 ± 5.5 nm in length and 5.3 ± 3.1 nm in width were selected as a representative system. The resulting Au NrCs exhibit L‐LSPR around 800 nm, which falls within the NIR‐I window, demonstrating that the self‐assembly process enables the formation of NIR‐responsive nanocapsules. To evaluate the generality of this system, six types of mini‐Au NRs with different ARs were subjected to the same assembly process. While the L‐LSPR positions of individual nanorods remained nearly unchanged after ligand modification and salt addition (Figures S8 and S9), consistent red shifts were observed after assembly in all cases. HAADF‐STEM images (Figure S10) confirm that capsule structures are successfully formed regardless of AR. These results demonstrate that the optical properties of Au NrCs can be effectively tuned by controlling the AR of the building blocks, enabling flexible design of plasmonic nanocapsules with resonance wavelengths spanning the visible to NIR regions while maintaining capsule sizes well within the range suitable for drug delivery applications.

### 2.3 Cargo Encapsulation into Au NrCs

Cargo encapsulation occurs concurrently with the formation of Au NrCs, driven by the same phase separation and concentration process described above. To visualize the encapsulation behavior, Au nanospheres (NSs) of different sizes and Ag NSs were employed as model cargoes. The Au NSs were purchased from BBI Solutions, while Ag NSs with an average diameter of 25 nm were synthesized in‐house (Figure S11). Citrate‐capped particles were functionalized with MDPA (12‐mercaptododecylphosphonic acid) to maintain their hydrophilic character during the assembly process (Figure 3a). Mini‐Au NRs, cargo nanoparticles, and citrate are co‐confined within aqueous domains that progressively concentrate in the organic solvent. Consistent with our previous study, this process enables simultaneous concentration and encapsulation of guest materials [50, 51]. Amphiphilic mini‐Au NRs preferentially migrate to and accumulate at the liquid–liquid interface, forming a particulate shell, whereas the hydrophilic MDPA‐modified NSs remain within the interior of the droplets.

**FIGURE 3:**
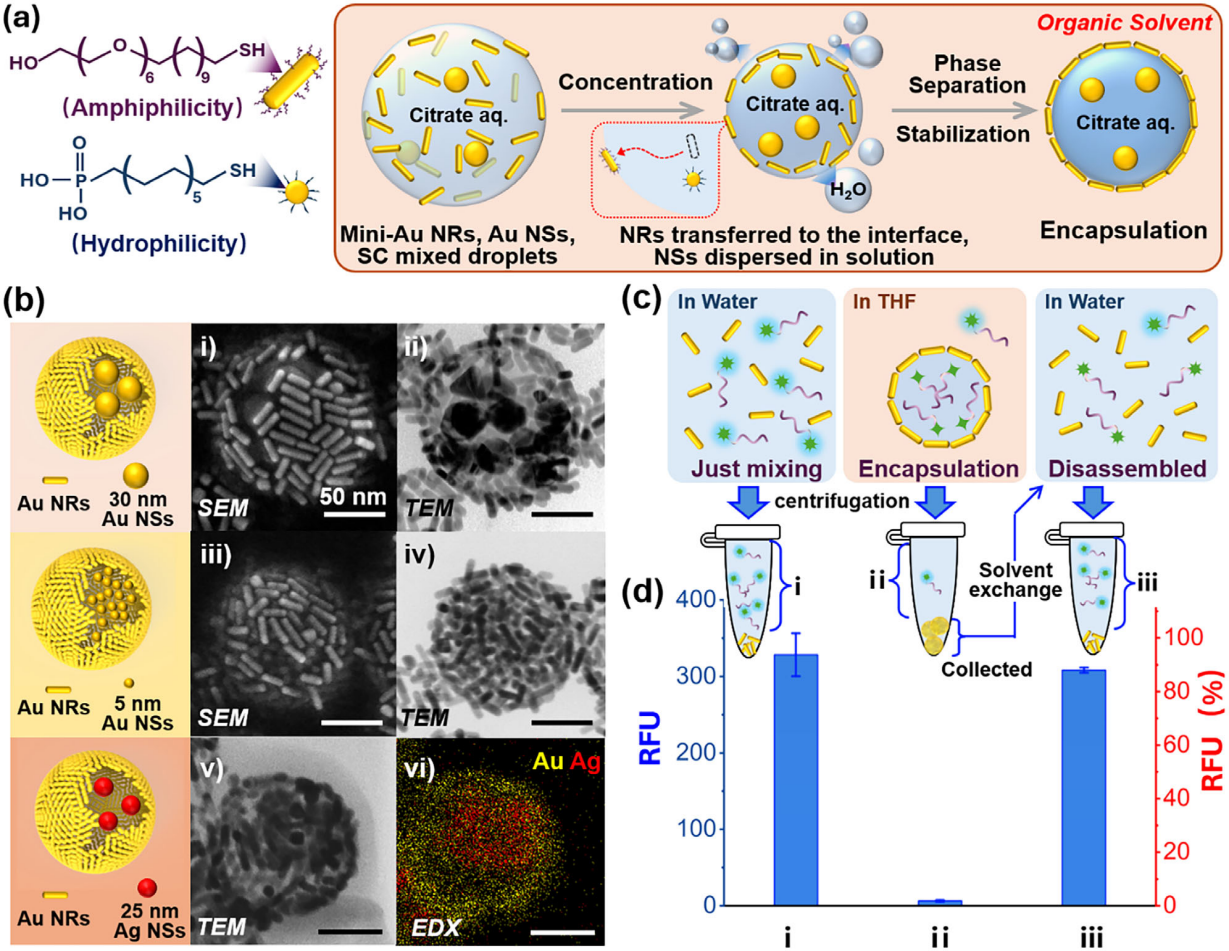
(a) Schematic illustration of cargo encapsulation by Au NrCs. (b) Electron microscopy images of Au NrCs encapsulating different nanoparticles: SEM (i) and TEM (ii) images for 30 nm Au NSs; SEM (iii) and TEM (iv) images for 5 nm Au NSs; TEM (v) and EDX elemental mapping (vi) images for 25 nm Ag NSs, where Au and Ag are shown in yellow and red, respectively. All scale bars are 50 nm. (c) Schematic illustration of the encapsulation of FAM‐DNA (57‐base) within Au NrCs and the evaluation procedure. (d) Fluorescence analysis of FAM‐DNA encapsulation: (i) Non‐adsorbed FAM‐DNA in the supernatant after centrifugation of a mixture of Au NRs and FAM‐DNA; (ii) Free (non‐encapsulated) FAM‐DNA remaining in the supernatant after capsule formation and centrifugation; (iii) Encapsulated FAM‐DNA recovered after disassembly of Au NrCs redispersed in 1 mL of water. The left (blue) y‐axis represents the fluorescence intensity at 520 nm (RFU), and the right (red) y‐axis shows the normalized fluorescence intensity relative to the total amount of FAM‐DNA used for the encapsulation experiment (defined as 100%). Data are presented as mean ± SD (*n* = 3).

SEM observations (Figure 3b) show that the capsule surfaces are densely covered with mini‐Au NRs, while cracks in the shell reveal encapsulated nanoparticles inside, including 30 nm Au NSs (Figure 3b‐i) and 5 nm Au NSs (Figure 3b‐iii). TEM and HAADF‐STEM images further confirm well‐defined capsule structures, with both 30 nm (Figure 3b‐ii, Figure S12) and 5 nm Au NSs (Figure 3b‐iv, Figure S13) clearly localized within the interior. Previous studies have reported size‐dependent segregation when nanoparticles with identical surface ligands are used, often resulting in yolk–shell or double‐shell structures [51, 54]. In contrast, the present system shows that encapsulation is governed primarily by ligand‐controlled amphiphilicity and hydrophilicity, rather than particle size. To further validate encapsulation and exclude potential artifacts associated with using the same material, Ag NSs were employed as alternative cargoes. Similar structural features were observed in SEM, TEM, and HAADF‐STEM images (Figure 3b‐v and Figure S14). Elemental mapping by energy‐dispersive X‐ray spectroscopy (EDX) shows that Ag (red) is localized within the interior, while Au (yellow) forms the outer shell (Figure 3b‐vi). The corresponding EDX spectra further confirm the presence of both elements (Figure S15). These results demonstrate that encapsulation is independent of nanoparticle composition and is instead governed by surface chemistry.

In addition to nanoparticle cargoes, this strategy is applicable to molecular species. FAM‐labeled DNA was selected as a model to evaluate encapsulation efficiency. As shown in Figure 3c, the encapsulation procedure was identical to that used for nanoparticles, and fluorescence measurements were used for quantification. Before fluorescence measurements, the Au NRs and Au NrCs were removed by centrifugation to eliminate fluorescence quenching. The encapsulation efficiency was calculated using the total amount of DNA as the reference (see Supporting Information for details). More than 90% of the total DNA was associated with the Au NrCs, while only approximately 8.6% of the total DNA was adsorbed onto the Au NR surfaces (Figure S16). Upon capsule disassembly, approximately 88% of the total DNA was recovered from the Au NrCs (Figure 3d), demonstrating efficient DNA encapsulation together with high release potential. These results are consistent with our previous findings and suggest that a similar solute‐induced concentration mechanism operates in the present system [50].

To estimate the degree of concentration enrichment, the enrichment factor was first estimated from TEM analysis of encapsulated Au NSs. Statistical analysis of the number of encapsulated Au NSs per capsule together with the known initial particle concentration yielded an estimated enrichment factor of approximately 1700‐fold (see for calculation details, Figure S17). This TEM‐based estimate supports substantial concentration enrichment during capsule formation. Additional estimations based on the citrate concentration required to induce solute‐driven phase separation and the capsule geometry also suggested enrichment factors of over 1000‐fold, providing further support for this estimated enrichment factor (see Supporting Information for calculation details) [50]. Taken together, these estimates support substantial concentration enrichment (>1000‐fold) within the capsule interior. Because a 25 µm DNA solution was used for encapsulation, the DNA concentration within the capsule interior is estimated to reach the order of several tens of millimolar. This extremely high local concentration highlights the ability of Au NrCs to enrich hydrophilic molecular cargoes without relying on strong electrostatic carrier–cargo interactions that could hinder efficient cargo release. Unlike conventional nucleic acid encapsulation strategies based on electrostatic condensation with cationic carriers, the present system enriches DNA through solute‐induced concentration during capsule formation. Such concentration‐assisted encapsulation may be advantageous for efficient cargo release upon capsule disassembly. These results demonstrate that Au NrCs enable efficient encapsulation and enrichment of guest materials, highlighting their potential as concentration‐assisted nanocarriers for biomolecular cargo delivery.

### 2.4 Surface Crosslinking of Au NrCs for Stable Dispersion in Water

Au NrCs prepared in organic solvents are inherently unstable in aqueous environments and rapidly disassemble into individual Au NRs upon transfer into water (Figure 4a‐ii), limiting their applicability, particularly in DDS [50]. To overcome this limitation, the as‐prepared Au NrCs were subjected to surface crosslinking to stabilize their structure in aqueous media. A bifunctional PEG linker terminated with epoxy groups was employed for crosslinking. Hydroxyl‐terminated EG6 ligands (EG6‐OH) exhibit limited reactivity toward epoxy groups, resulting in insufficient crosslinking (Figure S18). Therefore, amino‐terminated EG6 ligands (EG6‐NH_2_) were introduced in place of a portion of the EG6‐OH ligands to provide reactive sites for covalent crosslinking. As illustrated in Figure 4a and Figure S19, amino groups react with epoxy groups in organic solvents via nucleophilic ring‐opening, forming covalent β‐hydroxyamine linkages. These covalent bonds enable interparticle crosslinking, thereby stabilizing the capsule structure during transfer into water. An additional consideration was that the introduction of EG6‐NH_2_ increased the tendency of mini‐Au NRs to aggregate upon addition of sodium citrate, likely because of electrostatic interactions involving protonated amino groups. To suppress this behavior, NaOH aqueous solution was added prior to assembly. This treatment also enhanced the nucleophilicity of the amino groups, thereby facilitating the epoxy ring‐opening reaction (Figure S20). Zeta potential measurements supported this, showing a shift from approximately +20 mV to −3.5 mV, consistent with deprotonation (Figure S21) [55, 56]. Notably, this pH adjustment was limited to the assembly and crosslinking steps, and the resulting Au NrCs were subsequently stable in near‐neutral aqueous media, with their zeta potential remaining largely unaffected by the later addition of sodium citrate (Figure S21).

**FIGURE 4:**
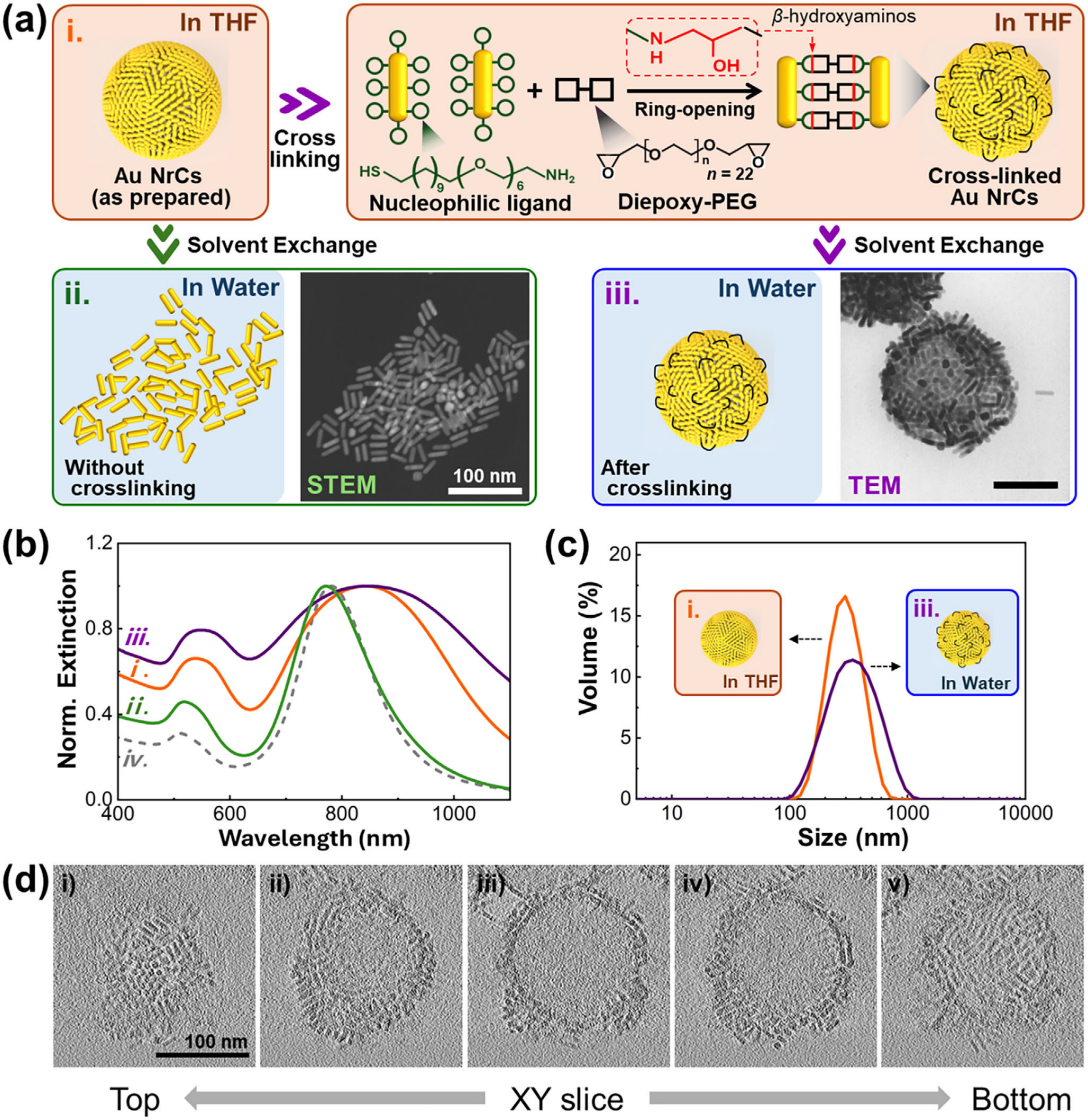
(a) Schematic illustration of solvent transfer of Au NrCs with and without crosslinking. (i) Crosslinking reaction scheme; (ii) uncross‐linked Au NrCs directly redispersed in water; (iii) cross‐linked Au NrCs redispersed in water. Scale bars: 100 nm. (b) Normalized extinction spectra of Au NrCs formed in THF (i, orange), uncross‐linked Au NrCs redispersed in water (ii, green), cross‐linked Au NrCs redispersed in water (iii, purple), and mini‐Au NRs (iv, grey), normalized at the L‐LSPR peak. (c) DLS profiles of cross‐linked Au NrCs dispersed in THF (orange) and water (purple). (d) Reconstructed XY‐plane images of cross‐linked Au NrCs in water obtained by cryo‐ET.

The modification ratio of EG6‐NH_2_ ligands was then systematically optimized to achieve an appropriate crosslinking density (Figure S22). Au NrCs modified with 5%–10% EG6‐NH_2_ were not sufficiently stabilized in water, whereas those modified with 50% EG6‐NH_2_ exhibited aggregation. Therefore, 30% EG6‐NH_2_ ligands were selected as the optimal composition for subsequent experiments. To further verify the role of interparticle crosslinking, a control experiment was performed using PEG with non‐reactive hydroxyl terminal groups instead of the diepoxy‐PEG crosslinker. Upon transfer into water, the Au NrCs disassembled, as confirmed by extinction spectra, DLS, and TEM measurements (Figure S23), supporting the conclusion that covalent interparticle crosslinking is essential for maintaining the capsule structure in aqueous media.

The structural stability of Au NrCs in aqueous media was evaluated after the crosslinking treatment by dispersing the samples in water. As shown in Figure 4b, uncross‐linked Au NrCs exhibited a pronounced blue shift upon redispersion (ii, green), indicating disassembly into individual nanorods. In contrast, cross‐linked Au NrCs retained spectral features similar to those observed in THF (iii, purple), with only slight peak broadening, indicating preservation of the assembled structure. Consistent with this result, DLS measurements show similar size distributions for cross‐linked Au NrCs in THF and water, confirming that the overall capsule size is maintained and that inter‐capsule aggregation is negligible (Figure 4c). TEM observations further support the retention of capsule morphology after transfer into water (Figure 4a‐iii). Additional structural characterization by SEM, TEM, and cryo‐TEM confirms the stability of the cross‐linked Au NrCs. SEM images show that the capsule morphology is preserved after crosslinking, with surfaces appearing coated by an organic layer (Figure S24d). TEM images indicate that the hollow capsule structure is maintained before and after crosslinking (Figure 4a‐iii and Figure S24c). Furthermore, 3D reconstructions obtained by cryo‐electron tomography (ET) clearly reveal hollow spherical shells in aqueous dispersion (Figure 4d). The corresponding tilt series and reconstructed videos are provided as Videos S1 and S2. Although a minor fraction of nanorod assemblies forms bundle‐like structures, the majority retain well‐defined capsule architectures. Notably, the cross‐linked Au NrCs retained capsule structure under biologically relevant conditions, including PBS, cell culture medium (DMEM supplemented with 10% FBS), and FBS, as confirmed by UV–Vis‐NIR extinction spectroscopy, DLS, and TEM (Figure S25). The Au NrCs exhibited excellent colloidal stability in PBS, whereas DLS measurements in the cell culture medium showed a predominant particle population with a hydrodynamic diameter of approximately 200 nm together with a small population of larger aggregates. TEM observations confirmed that the capsule structure was largely preserved after incubation in both PBS and the cell culture medium. These findings demonstrate that the cross‐linked Au NrCs maintain their structural integrity under biologically relevant conditions, providing an important basis for future biological investigations.

These results demonstrate that surface crosslinking effectively suppresses capsule disassembly in aqueous environments while preserving the hollow Au NrC structure, enabling their use in aqueous‐phase applications. In addition, the PEG‐derived surface hydrophilicity introduced through the ring‐opening reaction is also expected to contribute to the colloidal dispersibility of the Au NrCs in water.

### 2.5 NIR‐Responsive Behavior of Au NrCs

To investigate the NIR‐responsive behavior of Au NrCs, their photothermal performance and structural stability under NIR irradiation were evaluated (Figure 5a). The LSPR peak of Au NrCs is located at 810–850 nm; therefore, an 810 nm NIR LED was used as the irradiation source. Under continuous irradiation (120 mW cm^−2^), the temperature increased rapidly within the first 2 min to approximately 37°C–38°C and gradually approached a steady state at around 43°C, indicating efficient photothermal conversion (Figure 5b; Figures S26 and S27). The pronounced photothermal effect suggests that NIR irradiation may induce structural changes in Au NrCs [47]. As shown in Figure 5c, the L‐LSPR peak of Au NrCs, initially shifted to 810–850 nm after assembly, shifted back toward shorter wavelengths upon NIR irradiation, reaching approximately 770 nm. Compared with monodispersed mini‐Au NRs (780 nm), the irradiated samples still exhibit a slight blue shift, indicating that the Au NrCs do not fully disassemble into individual nanorods but instead form partially associated structures, likely constrained by crosslinking. Consistent with this interpretation, DLS measurements show that the hydrodynamic size distribution broadened significantly after irradiation, with the polydispersity index (PDI) increasing from 0.124 to 0.346, indicating increased structural heterogeneity (Figure S28). TEM observations further confirm partial disassembly and the formation of side‐by‐side aligned nanorod assemblies (Figure 5d,e; Figure S29). Control experiments performed by heating Au NrCs in a 45°C water bath for 30 min resulted in no significant changes in extinction spectra, size distribution, or morphology, indicating that bulk heating alone is insufficient to induce structural disassembly (Figure S30). These results suggest that the observed structural changes originate from localized photothermal effects generated by plasmonic coupling between adjacent nanorods.

**FIGURE 5:**
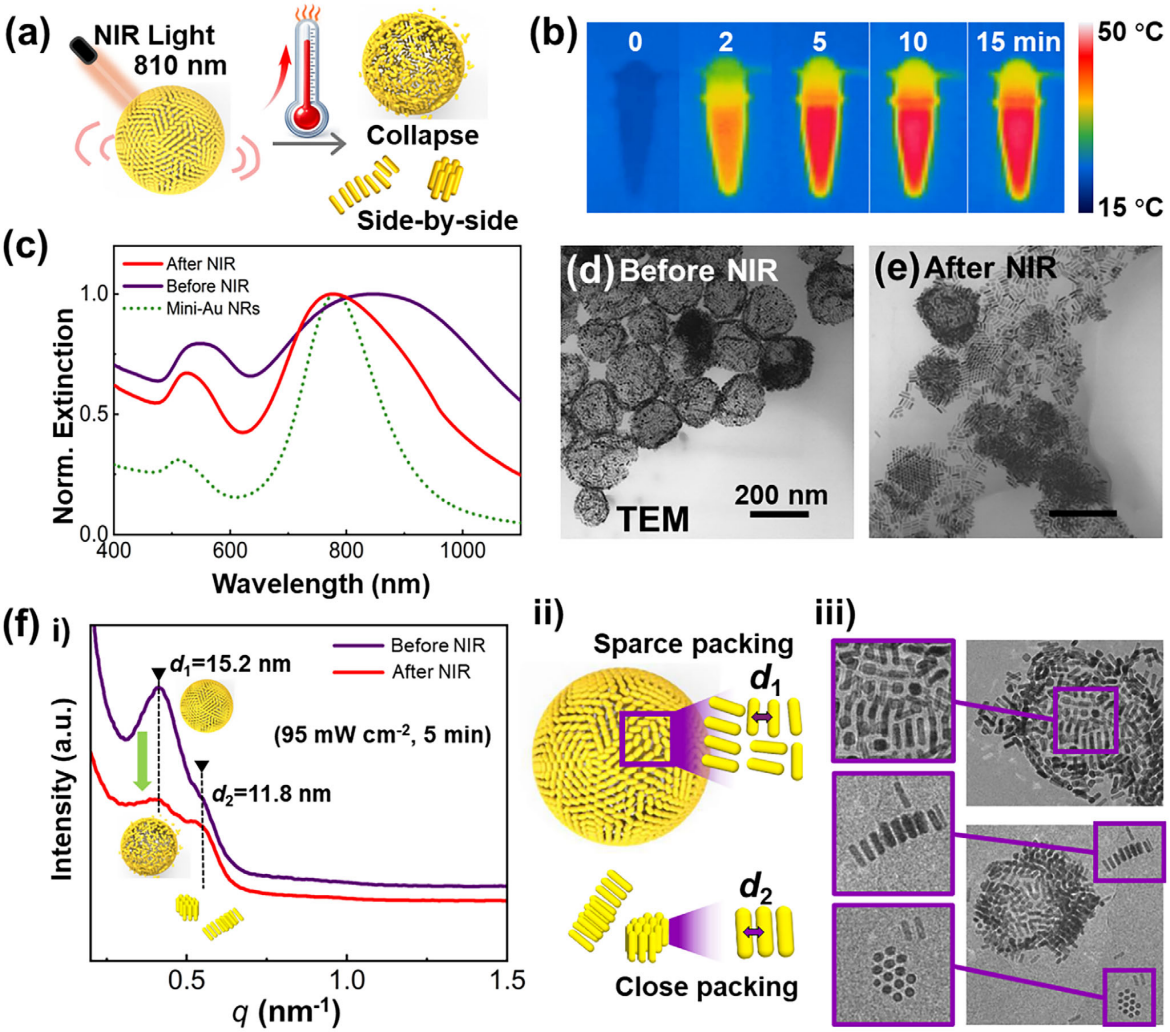
(a) Schematic illustration of the NIR‐responsive behavior of Au NrCs under irradiation. (b) Infrared thermal images of Au NrCs under 810 nm NIR irradiation (120 mW cm^−2^) at different times. (c) Normalized extinction spectra of mini‐Au NRs (green), Au NrCs before NIR irradiation (purple), and Au NrCs after NIR irradiation (red). (d,e) TEM image of Au NrCs dispersed in water before (d) and after (e) NIR irradiation at 120 mW cm^−2^. Scale bars are identical. (f) Structural analysis of Au NrCs before (red) and after (blue) NIR irradiation: (i) SAXS profiles; (ii) schematic interpretation; (iii) representative cryo‐TEM images.

Notably, these structural responses were induced using relatively low‐power NIR LED irradiation rather than the high‐power laser irradiation commonly employed in photothermal systems. To further examine the structural response, the effect of irradiation power density was investigated. Au NrCs retained their vesicular structure at power densities below 70 mW cm^−2^, whereas partial disruption was observed at 95 mW cm^−2^, and pronounced collapse occurred at higher intensities, demonstrating a clear power‐dependent structural evolution (Figure S31). Furthermore, extinction spectra and TEM observations showed that the Au NRs remained structurally stable under the NIR LED irradiation conditions employed in this study (Figure S32). Unlike the morphological changes reported under prolonged high‐power laser irradiation [57], the present relatively mild NIR LED irradiation induced capsule disassembly while maintaining the morphology and optical properties of the constituent Au NRs.

Finally, small‐angle X‐ray scattering (SAXS) measurements were performed to confirm that the NIR‐induced structural collapse occurs in the dispersed state rather than being an artifact of the drying process during TEM sample preparation. As shown in Figure 5f, the SAXS profile before NIR irradiation exhibits two characteristic peaks corresponding to interparticle distance of, *d*_1_ = 15.2 nm and *d*_2_ = 11.8 nm. Based on the cryo‐TEM images (Figure 5f‐iii), *d*_1_ is attributed to the predominant NR arrangement on the Au NrCs, while *d*_2_ reflects a small fraction of side‐by‐side close‐packed assemblies or localized aggregates of mini‐Au NRs. The difference between *d*_1_ and *d*_2_ (≈3.4 nm) is comparable to the effective dimension of coiled PEG chains rather than the fully extended length of the diepoxy‐PEG linker (*n* = 22) [58, 59]. This suggests that PEG‐mediated crosslinking contributes to the predominant NR arrangement observed on the capsule surface, which exhibits a larger average interparticle spacing than the side‐by‐side close‐packed assemblies represented by *d*_2_. After NIR irradiation, the SAXS peak corresponding to *d*_1_ decreased significantly, indicating disruption of the capsule architecture. These results, consistent with the spectral analyses and TEM observations, confirm that structural collapse can be effectively triggered by relatively mild NIR irradiation in solution. The combination of this efficient encapsulation and light‐induced structural transformation is expected to provide a robust platform for the controlled release of encapsulated cargo.

### 2.6 NIR‐Triggered Cargo Release via Structural Disassembly of Au NrCs

Building on the power‐dependent structural disassembly of Au NrCs demonstrated in the preceding section (Figure S31), we next investigated their ability to release encapsulated cargo under NIR irradiation. To directly visualize the release of nanoscale cargoes, Au NrCs encapsulating Ag NSs were irradiated with an 810 nm NIR LED (120 mW cm^−2^). As shown in the normalized extinction spectra (Figure 6a), a pronounced blue shift of the L‐LSPR peak was observed after irradiation, consistent with the structural disassembly described above. In addition, a characteristic absorption peak of Ag NSs appeared at approximately 400 nm, indicating the presence of well‐dispersed Ag NSs in solution. TEM observations further confirm that NIR irradiation induces collapse of the capsule structure and release of the encapsulated nanoparticles (Figure 6b).

**FIGURE 6:**
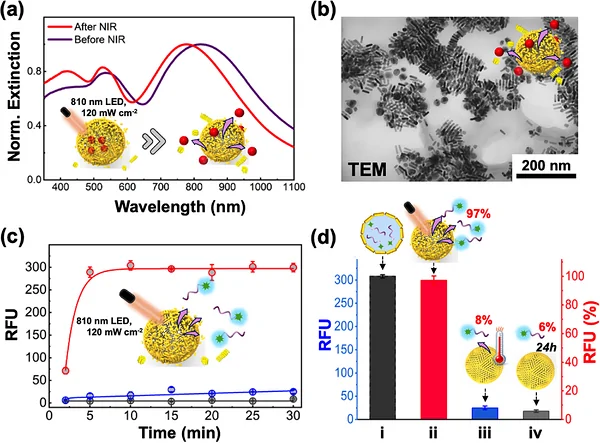
(a) Normalized extinction spectra of Ag NS‐loaded Au NrCs before and after NIR irradiation. (b) TEM image of Ag NS‐loaded Au NrCs after NIR irradiation (810 nm, 120 mW cm^−2^). (c) Time‐dependent fluorescence profiles of FAM‐DNA release: NIR irradiation (red), no irradiation (gray), and 45°C water bath (blue). (d) Quantitative analysis of DNA release under different conditions. Left axis: fluorescence intensity (RFU); right axis: percentage relative to total encapsulated DNA. (i) total encapsulated DNA (100%); (ii) released DNA after NIR irradiation; (iii) DNA released under 45°C water bath; (iv) DNA released after 24 h without irradiation at 25°C. Data are presented as mean ± SD (*n* = 3).

To quantitatively evaluate cargo release, FAM‐labeled DNA was used as a molecular cargo. The DNA‐loaded Au NrCs were first cross‐linked and then transferred into water for the light‐triggered release experiments. As shown in Figure 6c, the irradiated sample exhibited a rapid increase in fluorescence intensity within the first 5 min, followed by a plateau, indicating efficient release. In contrast, the non‐irradiated control showed negligible leakage, demonstrating that the cross‐linked Au NrCs effectively retain encapsulated DNA in the absence of NIR irradiation while enabling rapid release upon capsule disassembly. Based on the encapsulated DNA amount determined in Figure 3d, the release efficiency was estimated to be approximately 97%, indicating nearly complete release of the encapsulated DNA (Figure 6d). The extinction spectra (Figure S33a) and DLS results (Figure S33b) of DNA‐loaded Au NrCs showed behavior similar to that of Au NrCs without DNA, indicating that the presence of cargo does not significantly affect the NIR‐responsive structural changes.

To clarify the mechanism of release, control experiments were performed under bulk heating conditions. When samples were incubated in a 45°C water bath for 30 min, only ∼8% of the DNA was released (Figure 6c, d), indicating that bulk heating alone is insufficient to induce release. In addition, capsules composed of spherical Au NPs (Au NsCs), which exhibit negligible absorption at 810 nm, retained their capsule structures even after 30 min of NIR irradiation and showed only negligible DNA release, indicating that LSPR absorption by the Au NRs is essential for structural collapse and cargo release (Figure S34). These observations are consistent with a mechanism in which plasmonic excitation of adjacent Au NRs induces structural disassembly, leading to cargo release. In addition, the concentrated internal environment generated during capsule formation may facilitate rapid cargo diffusion and release following structural disassembly while minimizing spontaneous leakage under non‐irradiated conditions. Indeed, minimal DNA leakage (∼6%) was observed over 24 h at 25°C, indicating good structural stability of Au NrCs under non‐stimulated conditions (Figure 6d and Figure S35).

Together, these results demonstrate that Au NrCs enable efficient encapsulation and low‐leakage retention of cargoes together with efficient NIR‐triggered release under relatively mild LED irradiation conditions. Building on our previously established concentration‐assisted encapsulation strategy, the present Au NrCs integrate aqueous stabilization and NIR‐responsive cargo release within a single plasmonic nanocarrier platform, thereby extending its applicability toward nucleic acid delivery. The additional stability experiments further demonstrated that the capsule structures were largely preserved under biologically relevant conditions, although limited aggregation was observed in protein‐containing media. These findings suggest that the present platform provides a promising basis for future biological investigations while also indicating that further optimization of colloidal stability under complex biological environments will be desirable. In addition, comprehensive evaluation of long‐term colloidal stability, biosafety, cellular uptake, intracellular cargo release, and the long‐term *in vivo* fate of the Au NrCs will be important subjects for future studies before biomedical applications can be fully established.

## 3 Conclusion

In this study, we developed water‐dispersible and NIR‐responsive Au NrCs through a solute‐induced phase separation strategy using mini‐Au NRs and amphiphilic EG6 ligands. By tuning the Au NR ARs, the optical response of the capsules was systematically adjusted from the visible to the NIR region. The resulting hollow nanocapsules enabled efficient encapsulation and enrichment of both nanoparticle and molecular cargoes during capsule formation. Furthermore, surface crosslinking using amino‐functionalized ligands and diepoxy‐PEG significantly improved the aqueous stability and dispersibility of the Au NrCs, overcoming the instability of previously reported systems. The Au NrCs also exhibited efficient cargo release under low‐power 810 nm NIR LED irradiation through capsule disassembly, demonstrating the potential of plasmonic nanocapsules for externally controlled delivery under relatively mild NIR LED irradiation conditions. While the irradiance employed here was lower than that used in many previously reported laser‐triggered drug release systems with demonstrated biocompatibility, further studies are needed to evaluate the biological safety of the present Au NrCs, particularly with respect to localized photothermal damage around the Au NRs. Collectively, this work extends our previously established concentration‐assisted encapsulation strategy to water‐dispersible plasmonic nanocapsules by integrating aqueous stabilization with controllable NIR‐triggered cargo release. This integrated platform addresses key limitations of previously reported plasmonic nanocarriers and provides a promising basis for future externally controlled delivery applications.

## Funding

The Japan Society for the Promotion of Science (JSPS) KAKENHI (Grant Nos. JP24K01275, JP25K22884, and JP24K03258), a Grant‐in‐aid for JSPS Fellows (JP23KJ0010).

## Conflicts of Interest

The authors declare no conflicts of interest.

## Supporting information




**Supporting File 1**: smll75754‐sup‐0001‐SuppMat.docx.

**Supporting File 2**: smll75754‐sup‐0002‐VideoS1.mp4.

**Supporting File 3**: smll75754‐sup‐0003‐VideoS2.mp4.

## Data Availability

The data that support the findings of this study are available in the supplementary material of this article.
