# Supplementary material for: NIR‐Responsive Gold Nanorod Capsules for Efficient Cargo Encapsulation and Release
Source: Small. 2026 Sep 23;22(62):e75754. doi: 10.1002/smll.75754 (PMC13742900; doi:10.1002/smll.75754)
Supplement: Supplementary file 1 [file SMLL-22-e75754-s001.docx]

Supporting Information

NIR-Responsive Gold Nanorod Capsules for Efficient Cargo Encapsulation and Release

Dongyu Zhang, Takehiro Yachi,* Daisuke Unabara, Tasuku Hamaguchi, Koji Yonekura,

Kiyoshi Kanie, Kuniharu Ijiro, Hideyuki Mitomo*

1. Experimental

**Chemicals and apparatus.** L-Ascorbic acid, sodium borohydride (NaBH_4_), *n*-decanol, 12-mercaptododecylphosphonic acid (MDPA), hydrochloric acid (HCl), dulbecco's modified Eagle's medium (DMEM), fetal bovine Serum (FBS), phosphate-buffered saline (PBS), and polyethylene glycol (PEG, *M*_n_ ≈ 1000) were purchased from Sigma-Aldrich. Cetyltrimethylammonium bromide (CTAB), silver nitrate (AgNO_3_), chloroauric acid trihydrate (HAuCl_4_·3H_2_O), 1,4-dioxane, stabilizer-free tetrahydrofuran (THF), sodium hydroxide (NaOH), and trisodium citrate anhydrous were purchased from FUJIFILM Wako Pure Chemical Corporation (Japan). 11-Mercaptoundecanol hexaethyleneglycol ether (OH-EG6-C11-SH) was purchased from Dojindo Laboratories (Japan). Poly(ethylene glycol) diglycidyl ether (PEGDGE, n ≈ 22) was purchased from TCI (Japan). 20-(9-Mercaptononyl)-3,6,9,12,15,18-hexaoxaeicosane-1-amine hydrochloride (NH_2_-EG6-C11-SH) was purchased from ProChimia Surfaces Sp. zo.o. (Sopot, Poland). FAM-DNA (a 57-base oligonucleotide labeled with fluorescein at the 5' end; sequence: 5'-[FAM]-GAC CGG GAA TCG AAC CCG GGC CGC GGC GGT GAG AGC GCC GAA TCC TAA CCA CTA GAC-3') was purchased from Integrated DNA Technologies (USA). Citrate-capped Au nanospheres (5 nm and 30 nm) were purchased from BBI Solutions (UK). All reagents were used as received without further purification. Milli-Q water was used in all experiments. Scanning transmission electron microscopy (STEM) observations were performed using an HD-2000 system (Hitachi High-Tech, Japan) operated at an acceleration voltage of 200 kV. Extinction spectra were recorded using V-730 and V-770 UV–Vis–NIR spectrometers (JASCO, Japan). Dynamic light scattering (DLS) measurements were carried out using a Zetasizer Nano ZS (Malvern Panalytical, UK). Cryo-transmission electron microscopy (cryo-TEM) observations were conducted on a CRYO ARM^TM^ 300 II system (JEOL, Japan) operated at 300 kV. Fluorescence measurements were performed using a NanoDrop 3300 fluorospectrometer (Thermo Fisher Scientific, USA).

**Synthesis of Mini-Au Nanorods (Mini-Au NRs).** Mini-Au NRs were synthesized via a modified seed-mediated growth method following González-Rubio et al.^[52]^ The synthesis comprised three steps: (i) synthesis of Au seed, (ii) secondary growth of seeds, and (iii) anisotropic growth to yield mini-Au NRs.

**Synthesis of Au seed.** The growth solution was prepared by dissolving CTAB (9.11 g, 50 mm) and *n*-decanol (870 mg, 11 mm) in 500 mL of water, followed by stirring at 60 °C for 30–60 min. The solution was then cooled to 30 °C before use. Subsequently, 250 µL of 0.05 M HAuCl_4_ solution was added to 25 mL of the *n*-decanol/CTAB solution in a 50 mL glass vial and stirred at 300 rpm for 5 min. After the addition of 125 µL of 0.1 m ascorbic acid, the solution gradually changed from yellow to colorless. Freshly prepared NaBH_4_ solution (250 µL, 80 mm) was then rapidly injected under vigorous stirring (300 rpm). The resulting seed solution was aged at 30 °C for at least 60 min before use.

**Secondary growth of seeds.** Secondary growth was performed using the Au seeds obtained as described above. An aqueous CTAB solution containing n-decanol (15 mL, 50 mm CTAB, 11 mm n-decanol) was first transferred into a 50 mL glass vial. Subsequently, 150 µL of 0.05 m HAuCl_4_ solution was added, and the mixture was stirred at 300 rpm for 5 min in a water bath maintained at 30 °C. After homogenization, 77 µL of 0.1 m ascorbic acid was introduced. Then, 10 mL of the previously prepared seed solution was added to the secondary growth solution, after which stirring was stopped. Under these conditions, the ascorbic acid present in the growth solution reduced only 5% of the Au precursor onto the existing seeds. Therefore, after 90 min, an additional 11.6 µL of 0.1 m ascorbic acid was added under stirring (300 rpm), and the reaction was then allowed to proceed undisturbed for another 90 min. This step was repeated twice to ensure complete reduction of the gold precursor, resulting in a total addition of 112 µL of 0.1 m ascorbic acid (77 + 11.6 + 11.6 + 11.8 µL).

**Synthesis of Mini-Au NRs.** Mini-Au NRs were synthesized via a seed-mediated growth method. AgNO_3_ (150 µL, 0.01 m), HCl (350 µL, 1 m), HAuCl_4_ (100 µL, 0.05 m), and ascorbic acid (100 µL, 0.1 m) were sequentially added to 5 mL of freshly prepared *n*-decanol/CTAB aq. solution (11 mm /50 mm) in a 13.5 mL glass vial maintained at 30 °C under stirring. Subsequently, 5 mL of the previously prepared seed solution, consisting of 2.5 mL of the secondary-growth Au seed solution and 2.5 mL of *n*-decanol/CTAB mixture, was introduced into the growth solution under vigorous stirring (1050 rpm). After 30 s, stirring was stopped, and the Au NRs were allowed to grow undisturbed at 30 °C for 3 h. The resulting mini-Au NRs were collected by centrifugation at 20100 × g for 120 min using 2 mL Eppendorf tubes. The precipitate was redispersed in 4 mL of 4 mm CTAB solution, yielding a final Au^0^ concentration of 5–10 mm. The obtained mini-Au NRs were stored at 30 °C for further use.

**Synthesis of Au NRs with Different Aspect Ratios.** Au NRs with different aspect ratios (ARs) were prepared by varying the amount of AgNO_3_ added during the growth process.^[53]^ Specifically, different volumes of 0.01 M AgNO_3_ solution (50, 75, 100, 150, 250, and 350 µL), together with HCl (350 µL, 1 m), HAuCl_4_ (100 µL, 0.05 m), and ascorbic acid (100 µL, 0.1 m), were sequentially added to 5 mL of freshly prepared *n*-decanol/CTAB aq. solution (11 mm/50 mm) in a 13.5 mL glass vial maintained at 30 °C under magnetic stirring. Subsequently, 5 mL of the secondary-growth Au seed solution was introduced into the growth solution under vigorous stirring (1050 rpm). After 30 s, stirring was stopped, and the Au NRs were allowed to grow undisturbed at 30 °C for 3 h. The purification and storage procedures were identical to those described above.

**Synthesis of Au NRs with Different Sizes.** Au NRs with different sizes were obtained by varying the volume of seed solution added to the growth system. AgNO_3_ (150 µL, 0.01 m), HCl (350 µL, 1 m), HAuCl_4_ (100 µL, 0.05 m), and ascorbic acid (100 µL, 0.1 m) were sequentially added to 5 mL of freshly prepared *n*-decanol/CTAB aq. solution (11 mm/50 mm) maintained at 30 °C under magnetic stirring. Subsequently, different volumes of the seed solution (0.1, 0.2, 0.5, 1, 2, 4, and 6 mL) were introduced into the growth solution under vigorous stirring (1050 rpm). After 30 s, stirring was stopped, and the Au NRs were allowed to grow undisturbed at 30 °C for 3 h. The purification and storage procedures were identical to those described above.

**Self-Assembly of Mini-Au NRs into Au NrCs.** Mini-Au NRs dispersed in 4 mm CTAB solution (300 µL) were transferred into a 1.5 mL tube and centrifuged at 20,100 × g for 40 min. The supernatant was removed, leaving 25 µL of concentrated mini-Au NRs. The NRs were then redispersed in 975 µL 0.5 mm EG6-OH solution and shaken at 150 rpm for 8 h to allow ligand exchange. After ligand exchange, the mixture was centrifuged again, and the supernatant was removed to obtain 20 µL of concentrated mini-Au NRs. This washing step was repeated three times to completely remove excess EG6-OH ligands. The resulting mini-Au NRs were dried under vacuum. Subsequently, 15 µL of trisodium citrate solution (3 mm) was added. Finally, 15 µL of the EG6-OH modified Au NRs was rapidly injected into 985 µL of 1,4-dioxane, vortexed vigorously for 1 s, and then allowed to stand for 2 h to form a stable dispersion of Au NrCs. The final Au^0^ concentration was 4.77 × 10^-2^ mg mL^-1^.

**Encapsulation of Nanoparticles into Au NrCs.** Mini-Au NRs with a L-LSPR peak at 750 nm were used as building blocks for the formation of Au NrCs. The encapsulated nanoparticles included 5 and 30 nm Au NSs (BBI Solutions) and 25 nm Ag NSs. First, 300 µL of mini-Au NRs were modified with EG6-OH ligands following the ligand exchange procedure described above. After ligand exchange, the NRs were centrifuged and washed three times to remove excess ligands and then dried under vacuum. To ensure good colloidal stability and dispersibility in aqueous media, the NSs to be encapsulated were modified with MDPA. The modified 5 nm Au NSs, 30 nm Au NSs, and 25 nm Ag NSs were then dispersed in 3 mm trisodium citrate solution. The concentrations of the nanoparticle solutions were adjusted to 30 nm (5 nm Au NSs), 0.7 nm (30 nm Au NSs), and 0.9 nm (25 nm Ag NSs), respectively. For encapsulation, 15 µL of the nanoparticle solution (5 nm Au NSs, 30 nm Au NSs, or 25 nm Ag NSs) was added to the dried mini-Au NRs and mixed thoroughly. The mixture was then rapidly injected into 985 µL of 1,4-dioxane, vortexed vigorously for 1 s, and allowed to stand for 2 h to form Au NrCs containing the encapsulated NSs. For the preparation of Au NsCs, commercially available Au nanospheres (Au NSs, BBI Solutions) were used. The Au NSs were surface-modified using the same procedure and the same ligand ratio as those used for the Au NRs before self-assembly.

**Encapsulation of FAM-DNA into Au NrCs.** The encapsulation procedure for FAM-DNA was similar to that used for nanoparticle loading. Briefly, 300 µL of mini-Au NRs were first modified with EG6-OH ligands following the ligand exchange procedure described above. After ligand exchange, the NRs were centrifuged and washed three times to remove excess ligands, followed by drying under vacuum. Subsequently, 10 µL of 50 µm FAM-DNA was mixed with 10 µL of 3 mm trisodium citrate solution containing NaOH (3 mm). The resulting solution was added to the dried mini-Au NRs and thoroughly mixed. The mixture was then rapidly injected into 980 µL of THF, vortexed vigorously for 1 s, and allowed to stand for 2 h to form DNA-encapsulating Au NrCs, with a final theoretical DNA concentration of 0.5 µm. To establish a fluorescence reference for the total DNA amount, a control sample was prepared by dispersing the same amounts of Au NRs and DNA (0.5 µm) in 1 mL of water without capsule formation. After centrifugation of this control, the fluorescence intensity of DNA in the supernatant was measured and defined as the initial DNA concentration. For the evaluation of encapsulation efficiency, the sample was centrifuged, and the fluorescence intensity of DNA in the THF supernatant was measured to determine the amount of unencapsulated DNA. Subsequently, the collected Au NrCs were redispersed in 1 mL of water, leading to their disassembly into Au NRs. After centrifugation, the fluorescence intensity of DNA in the supernatant was measured to determine the amount of encapsulated DNA.

**Calculation of DNA Encapsulation Efficiency and Evaluation of DNA Adsorption onto Au NRs.** To define the total amount of DNA used for the encapsulation efficiency calculation, a DNA solution containing the same amount of FAM-DNA as that used in the encapsulation experiment was prepared in 1 mL of water. The fluorescence intensity of this solution was defined as the 100% reference for the encapsulation efficiency analysis. To evaluate DNA adsorption onto the Au NR surfaces under the encapsulation conditions, the same amount of FAM-DNA (0.5 μM) was mixed with EG6-modified Au NRs prepared under identical solution conditions to those used for the encapsulation experiment. After incubation, the Au NRs were removed by centrifugation, and the fluorescence intensity of the supernatant was measured. The amount of DNA adsorbed onto the Au NR surfaces was estimated according to

$$\mathrm{DNA}adsorption \left( \% \right)=\left( 1-\frac{F_{supernatant}}{F_{reference}} \right)\times100$$

where *F*_reference_ is the fluorescence intensity of the DNA solution without Au NRs, and *F*_supernatant_ is the fluorescence intensity of the supernatant obtained after centrifugation of the DNA/Au NR mixture.

**Surface Crosslinking and Dispersion of Au NrCs in Water.** To tune the L-LSPR peak of Au NrCs to 810 nm for subsequent NIR-responsive release experiments, mini-Au NRs with an L-LSPR peak at 780 nm were used as building blocks. Mini-Au NRs were modified with EG6 ligands according to the procedure described above, with EG6-NH_2_ accounting for 30% of the total ligand content. After ligand exchange and purification, the modified mini-Au NRs were dried under vacuum. Subsequently, a mixed solution of trisodium citrate and NaOH (1.66 mm, 20 µL) was added. The resulting EG6-modified mini-Au NRs (20 µL) were rapidly injected into 980 µL of THF, vortexed vigorously for 1 s, and then allowed to stand for 2 h to form a stable dispersion of Au NrCs. After assembly for 2 h, a 20 mm diepoxy-PEG solution in THF (100 µL) was added. The mixture was gently mixed and allowed to stand at room temperature for 8 h to complete the crosslinking reaction. After crosslinking, the sample was centrifuged at 500 × g for 15 min, and the precipitate was washed three times with THF to remove excess crosslinker, followed by redispersion in 1 mL of water. The surface crosslinking and transfer of Au NsCs into water were performed using the same procedure as that described for Au NrCs. To examine the effect of amino ligand content on the structural stability of Au NrCs after transfer into water, the proportion of EG6-NH_2_ was varied to 5%, 10%, or 50% of the total EG6 ligand concentration during ligand modification of the Au NRs. All subsequent procedures for capsule formation, surface crosslinking with diepoxy-PEG, and transfer into water were identical to those described above. For the control experiment using a non-reactive PEG linker, diepoxy-PEG was replaced with polyethylene glycol (PEG, Mn ≈ 1000) at the same concentration. All other experimental conditions were identical to those used for the standard surface crosslinking procedure.

**Evaluation of the Stability of Au NrCs under Biologically Relevant Conditions.** The stability of the cross-linked Au NrCs under biologically relevant conditions was evaluated in phosphate-buffered saline (PBS), cell culture medium (DMEM supplemented with 10% FBS), and FBS. DMEM (Low Glucose) was supplemented with 10% (v/v) heat-inactivated FBS to prepare the cell culture medium. After cross-linking, the Au NrCs were collected by centrifugation and the supernatant was removed, leaving approximately 20 µL of the concentrated Au NrC dispersion. Subsequently, 980 µL of PBS, cell culture medium, or FBS was added, and the samples were gently mixed and incubated at 37 °C for 2 h. The dispersibility and structural stability of the Au NrCs were then evaluated by UV-Vis-NIR spectroscopy and DLS. The morphology of the Au NrCs dispersed in PBS and cell culture medium was further examined by TEM. TEM characterization of the Au NrCs dispersed in FBS was not performed because the high protein concentration made reliable TEM observation difficult.

**Cryo-TEM Tomography.** For cryo-TEM observation, the Au NrCs dispersed in THF or water were applied to a glow-discharged holey carbon TEM grid (Quantifoil R0.6/1 or R1.2/1.3 Cu 200 mesh, Quantifoil Micro Tools GmbH, Germany). Excess solvent was removed via filter paper in a semi-automated vitrification plunger, EM GP2 (Leica, Germany), held at 4 °C, followed by rapid vitrification in liquid N_2_ for the THF dispersion without humidity control or liquid ethane for the water dispersion at 95% relative humidity. The water dispersion was sonicated before vitrification. Cryo-TEM and ET observations were conducted using a CRYO ARM 300II system (JEOL, Japan) operated at an acceleration voltage of 300 kV, with the specimen temperatures maintained at ~ 90 K. Zero-loss images were recorded using an in-column energy filter with a slit width of 20 eV at a nominal magnification of ×25,000 on a direct electron detector (AMETEK K3) in CDS mode. Tilt series were collected for the water dispersion via SerialEM^[60]^ using a custom-modified FastTomo script,^[61]^ employing a bidirectional tilt scheme from −60° to +60°, with 3° increments. The electron rate and exposure time per tilt angle were set to ~ 2.8 e⁻/Å^2^/s and ~ 0.7 s, respectively, corresponding to 1.9 e⁻/Å^2^ per image consisting of 5 dose-fractionated frames. The total accumulated dose for each tilt series was ~ 80 e⁻/Å^2^. For the THF dispersion, untilted images, each consisting of 20 frames, were collected over a 4 s exposure. Three-dimensional reconstructions were generated from the tilt series using the eTomo module within the IMOD software suite.^[62]^

**Estimation of the Enrichment Factor Based on TEM Analysis of Encapsulated Au Nanospheres.** The enrichment factor was estimated from TEM observations of Au nanospheres (Au NSs) encapsulated within Au NrCs. The average number of encapsulated Au NSs per Au NrC (*N_avg_*) was calculated as

$$N_{avg}=\frac{\sum_{i=1}^{n} N_{i}}{n}$$

where *N_i_* is the number of Au NSs observed in the *i*th capsule and *n* is the total number of analyzed capsules. Capsules without encapsulated Au NSs were included in the analysis to avoid overestimating the average number of encapsulated particles.

The Au NrCs were approximated as spheres with an average diameter of 120 nm, as determined from TEM measurements. The apparent capsule volume (*V*) was therefore estimated as

$$V=\frac{4}{3}\pi r^{3}$$

where *r* = 60 nm, giving

$$V=9.05\times{10}^{-19}L$$

The apparent Au NS concentration inside the Au NrCs (C*_capsule_*) was estimated as

$$C_{capsule}=\frac{N_{avg}}{N_{A}V}$$

where *N*_A_ is Avogadro's constant.

The enrichment factor (*EF*) was estimated by comparing the apparent Au NS concentration inside the Au NrCs with the initial Au NS concentration used for the encapsulation experiment:

$$EF=\frac{C_{capsule}}{C_{0}}$$

where *C_0_* is the initial Au NS concentration used for the encapsulation experiment.

From TEM analysis, the average number of encapsulated Au NSs was estimated to be 4.7 particles per Au NrC. Using an initial Au NS concentration of 5.0 nM, the apparent Au NS concentration inside the Au NrCs was estimated to be 8.7 μM, corresponding to an estimated enrichment factor of

$$EF\approx1.74\times{10}^{3}$$

Thus, the enrichment factor was estimated to be approximately 1700-fold relative to the initial Au NS concentration.

**Estimation of Internal Concentration Enrichment Based on Citrate Saturation.** In our previous study on plasmonic nanoparticle capsules formed through solute-induced phase separation, a sodium citrate concentration of 0.75 mm in the initial aqueous phase was estimated to be concentrated by approximately 2500-fold during capsule formation, reaching the concentration range close to citrate saturation and thereby inducing liquid–liquid phase separation ^[50]^. In the present system, the initial sodium citrate concentration was increased to 1.66 mm. Assuming that capsule formation similarly occurs when the local citrate concentration approaches the saturation range, the enrichment factor (*EF*_citrate_) in the present system was estimated according to:

$${EF}_{citrate}=\frac{2500 \times0.75}{1.66}$$

yielding an estimated enrichment factor of approximately 1100. This result suggests that the internal aqueous phase within the Au NrCs is concentrated on the order of 1000-fold during capsule formation.

2. Supporting Figures

**Figure S1.** Extinction spectra of Au seeds before (purple) and after (red) secondary growth. Insets show photographs of the Au seed solutions before and after growth.

**Figure S2.** (a–d) HAADF-STEM images corresponding to samples 2–5 in Figure 1b, respectively. Scale bars in panels (b–d) are identical to that in panel (a).

**Figure S3.** Length and width distributions of mini-Au NRs (samples 1-6) determined from TEM images using ImageJ. The average length and width are presented as mean ± SD, with the number of analyzed nanorods (n) indicated in each panel. The aspect ratio (AR) was calculated from the mean length and mean width of each sample.

**Figure S4.** Low-magnification HAADF-STEM image of Au NrCs.

**Figure S5.** Statistical analysis of the Au NrC size distribution from HAADF-STEM images. (a) Representative HAADF-STEM image used for the statistical analysis. Individual Au NrCs were manually identified and measured using ImageJ, as indicated by the numbered labels. (b) Size distribution of Au NrCs determined from the HAADF-STEM images. A total of 273 capsules were analyzed, yielding an average capsule diameter of 98.7 ± 19.0 nm (mean ± SD, *n* = 273).

**Figure S6.** A representative cryo-TEM image of Au NrCs dispersed in THF.

**Figure S7.** Batch-to-batch reproducibility of the Au NrCs prepared by the same fabrication protocol. (a) Normalized UV-Vis-NIR spectra of Au NrCs from three independently prepared batches. (b) DLS size distributions of the corresponding batches. The inset shows the polydispersity index (PDI) of each batch. (c) Representative TEM images of Au NrCs from (i) Batch 1, (ii) Batch 2, and (iii) Batch 3 (Scale bars: 100 nm). The nearly identical UV-Vis-NIR spectra, DLS profiles, and capsule morphologies demonstrate the high reproducibility of the present fabrication strategy.

**Figure S8.** Normalized extinction spectra of samples 1–6 at different stages: pristine mini-Au NRs before ligand modification (a), after ligand modification (b), after addition of trisodium citrate (c), and after self-assembly into Au NrCs (d). For clarity, the L-LSPR bands of Au NrCs and the T-LSPR bands of mini-Au NRs were normalized separately. Corresponding LSPR peak positions are indicated using matching colors.

**Figure S9.** Photographs of samples 1–6 before (left) and after (right) self-assembly of mini-Au NRs into Au NrCs.

**Figure S10.** HAADF-STEM images of Au NrCs formed by self-assembly of mini-Au NRs in dioxane (samples 1–6). Scale bars in panels (b–f) are identical to that in panel (a).

**Figure S11.** Characterization of Ag NSs: (a) extinction spectrum; (b) DLS hydrodynamic size distribution; (c) low-magnification HAADF-STEM image; and (d) high-magnification HAADF-STEM image.

**Figure S12.** HAADF-STEM image of Au NrCs encapsulating 30 nm Au nanospheres (Au NSs).

**Figure S13.** HAADF-STEM image of Au NrCs encapsulating 5 nm Au NSs.

**Figure S14**. HAADF-STEM, SEM, and TEM images of Au NrCs encapsulating 25 nm Ag NSs. All scale bars are 100 nm.

**Figure S15.** (a) TEM image of Au NrCs encapsulating 25 nm Ag NSs; (b) EDX elemental map of Ag; (c) EDX elemental map of Au; (d) overlay of Au and Ag elemental maps; and (e) EDX spectrum of Au NrCs encapsulating Ag NSs, showing characteristic Au and Ag signals. The Cu signal originates from the TEM grid.

**Figure S16.** Evaluation of DNA adsorption onto Au NRs under the encapsulation conditions. (a) Calibration curve of FAM-DNA fluorescence intensity (RFU) as a function of DNA concentration in water in the absence of Au NRs. (b) Fluorescence analysis of FAM-DNA solutions used to evaluate DNA adsorption onto Au NRs under standardized aqueous conditions. (i) FAM-DNA dispersed in water without Au NRs (defined as the 100% reference); (ii) supernatant obtained after centrifugation of a mixture of FAM-DNA and EG6-OH-modified Au NRs; and (iii) supernatant obtained after centrifugation of a mixture of FAM-DNA and Au NRs modified with EG6-OH and 30% EG6-NH_2_. The same amounts of FAM-DNA and Au NRs were used in (ii) and (iii). Comparison with (i) indicated that approximately 8.6% and 9.4% of the DNA was adsorbed onto the Au NRs in (ii) and (iii), respectively. Data are presented as mean ± SD (*n* = 3).

**Figure S17.** TEM-based statistical analysis used for estimation of the nanoparticle enrichment factor. (a) Frequency distribution of the number of encapsulated Au NSs per Au NrC. All analyzed Au NrCs, including capsules without encapsulated Au NSs, were included in the statistical analysis to avoid overestimating the average number of encapsulated particles. The average number of encapsulated Au NSs was 4.7 ± 2.8 NSs per capsule (mean ± SD, *n* = 54). (b) Size distribution of the NrCs determined from TEM images, showing an average diameter of 119.6 ± 21.9 nm (mean ± SD, *n* = 54). The average capsule diameter was used to estimate the apparent capsule volume for the enrichment factor calculation. (c) Representative TEM image of Au NrCs prepared in the presence of 20 nm Au NSs. Based on the statistical analysis in (a) and the average capsule volume in (b), the apparent Au NS concentration inside the Au NrCs was estimated to be 8.7 μM, corresponding to an estimated enrichment factor of approximately 1700-fold (see Calculation Method).

**Figure S18.** (a) Schematic illustration of partially cross-linked surfaces in Au NrCs assembled from Au NRs modified only with EG6-OH ligands. (b) Normalized extinction spectra of cross-linked Au NrCs prepared using only EG6-OH ligands and dispersed in THF (orange dashed line) and water (purple dashed line). Spectra were normalized at the L-LSPR peak. (c) DLS profiles of the corresponding cross-linked Au NrCs dispersed in THF (orange dashed line) and water (purple dashed line). (d,e) TEM images of cross-linked Au NrCs dispersed in THF (d) and water (e). Scale bars are identical.

**Figure S18** shows that mini-Au NRs modified only with EG6-OH ligands exhibit limited crosslinking efficiency under the same reaction conditions. As shown in panel (b), a pronounced blue shift in the extinction spectrum is observed after redispersion of the cross-linked Au NrCs in water, indicating partial structural disassembly. DLS analysis further reveals a significantly broadened size distribution ranging from 40 to 1000 nm. TEM observations show that the Au NRs on the surface of Au NrCs become sparsely distributed in water, with only faint remnants of the original capsule structure retained, while a large number of individually dispersed mini-Au NRs are also observed. These results suggest that insufficient crosslinking leads to partial disassembly of Au NRs from the capsule surface in aqueous media. Therefore, EG6-OH ligands alone are insufficient to maintain the structural integrity of Au NrCs in water, highlighting the importance of introducing more nucleophilic ligands to promote the epoxy ring-opening reaction and improve structural stability.

**Figure S19.** Chemical scheme of the epoxy ring-opening reaction used for surface crosslinking of Au NrCs.

**Figure S19** illustrates the ring-opening reaction between the diepoxy-PEG crosslinker and EG6-NH_2_ surface ligands. Upon deprotonation, the amino groups exhibit strong nucleophilicity and readily react with epoxide groups through nucleophilic ring-opening, forming secondary amine (β-hydroxyamine) structures with relatively high reaction efficiency. These secondary amines also retain further nucleophilic reactivity and can react with additional epoxide groups to form tertiary amine structures, although this secondary crosslinking process is relatively slower. Such reactions improve overall crosslinking while reducing the presence of residual free amine groups, thereby minimizing potential charge-related effects after crosslinking.

**Figure S20.** (a) Schematic illustration of sodium citrate-induced aggregation of amine-functionalized mini-Au NRs and its suppression by NaOH addition. (b) Schematic illustration of deprotonation of surface amino groups upon NaOH treatment.

**Figure S21.** Zeta potentials of EG6-OH-functionalized and EG6-OH/EG6-NH2-co-functionalized mini-Au NRs measured in water, 1.66 mm NaOH solution, and a mixed solution of 1.66 mm NaOH and sodium citrate.

**Figure S22.** Effect of NH_2_ ligand content on the crosslinking behavior and structural stability of Au NrCs. Au NrCs were prepared with NH_2_ ligand contents of 5% (a), 10% (b), and 50% (c), while all other experimental conditions were kept constant. Their structural stability after transfer to water was evaluated by UV-Vis-NIR extinction spectroscopy (i), DLS (ii), and TEM (iii). At an NH_2_ ligand content of 5%, a pronounced blue shift in the extinction spectra and the appearance of a 10–20 nm peak together with a broad peak at larger sizes in the DLS profile indicated structural instability after transfer to water. TEM observations confirmed extensive side-by-side aggregation of Au NRs, indicating insufficient crosslinking to maintain the capsule structure. Increasing the NH_2_ ligand content to 10% improved capsule retention, although partial disassembly and aggregation were still observed. In contrast, Au NrCs prepared with 50% NH_2_ ligands exhibited almost no spectral or size changes after transfer to water, and TEM confirmed that the capsule morphology was largely preserved. A small degree of aggregation was also observed. These results demonstrate that the NH_2_ ligand content strongly influences the structural stability of Au NrCs, indicating that an appropriate crosslinking density is required to achieve aqueous stability while avoiding aggregation.

**Figure S23.** Verification of the role of covalent crosslinking using a non-reactive PEG control. Hydroxyl-terminated PEG1000 was used as a non-reactive control instead of the diepoxy-PEG crosslinker. (a) Normalized UV-Vis-NIR extinction spectra and (b) DLS size distributions of Au NrCs dispersed in THF and after transfer to water. The extinction spectra reverted to a profile similar to that of dispersed mini-Au NRs, accompanied by the disappearance of the broad L-LSPR band characteristic of the capsule structures, while the DLS profiles shifted toward smaller particle sizes. (c, d) Representative TEM images of Au NrCs dispersed in (c) THF and (d) water. No intact capsule structures were observed after transfer to water, confirming that covalent crosslinking is essential for maintaining the capsule structure in aqueous media. Scale bars: 100 nm.

**Figure S24**. (a) HAADF-STEM image of mini-Au NRs before assembly; (b) SEM image and (c) TEM image of Au NrCs formed in THF; and (d) SEM image of cross-linked Au NrCs redispersed in water. All scale bars are 100 nm.

**Figure S25.** Stability of Au NrCs under biologically relevant conditions. Stability of the cross-linked Au NrCs after incubation for 2 h in (a) PBS, (b) cell culture medium (DMEM supplemented with 10% FBS), and (c) FBS. For each condition, (i) UV-Vis-NIR extinction spectra, (ii) DLS size distributions, and (iii) representative TEM images are shown. The Au NrCs remained highly stable in PBS, while incubation in DMEM supplemented with 10% FBS resulted in a predominant particle population with a hydrodynamic diameter of approximately 200 nm together with a small population of larger aggregates. TEM observations confirmed that the capsule structures were largely preserved after incubation in PBS and the cell culture medium. Reliable TEM observation in FBS was not feasible because of its high protein concentration.

**Figure S26.** Schematic illustration of the experimental setup for temperature monitoring under NIR irradiation. Surface temperature was measured using an infrared thermal imaging camera (a), while internal solution temperature was measured using a contact temperature sensor (b).

**Figure S27.** Internal solution temperature of Au NrCs and a blank control (water) as a function of NIR irradiation time. Data are presented as mean ± SD (*n* = 3).

**Figure S28.** DLS profiles of Au NrCs before and after NIR irradiation. The red profile corresponds to Au NrCs before irradiation (PDI = 0.124), while the blue profile corresponds to Au NrCs after irradiation (PDI = 0.346).

**Figure S29.** TEM image of Au NrCs after NIR irradiation. Structural changes induced by irradiation include collapsed Au NrCs (blue boxes), two-dimensional side-by-side assemblies of Au NRs (yellow boxes), and three-dimensional aggregates composed of side-by-side aligned Au NRs (red boxes).

**Figure S30**. (a) Normalized extinction spectra of Au NrCs before (purple) and after (blue) heating at 45 °C for 30 min, normalized at the L-LSPR peak. (b) DLS size distribution profiles of Au NrCs before (purple) and after (blue) heating at 45 °C for 30 min. (c) TEM image of Au NrCs after heating in water at 45 °C for 30 min.

Under 810 nm LED irradiation (120 mWcm^-2^), the sample solution temperature increased to a maximum of 43 ℃, corresponding to thermal equilibrium between the system and the surrounding environment. To clarify whether the structural disassembly of Au NrCs originates from localized photothermal effects induced by plasmonic coupling rather than bulk heating alone, control experiments were performed by incubating the samples in a 45 °C water bath, slightly above the maximum temperature reached under NIR irradiation. As shown in **Figure S30**, the extinction spectra of Au NrCs exhibited negligible changes after heating at 45 °C. Consistently, DLS measurements revealed no significant variation in particle size distribution following thermal treatment. TEM observations further confirmed that Au NrCs largely retained their spherical vesicular morphology after incubation at 45 °C for 30 min, with no obvious structural disassembly observed. These results indicate that bulk heating at 45 °C alone is insufficient to induce significant structural changes in Au NrCs. Therefore, the structural transformation observed under NIR irradiation is more likely attributed to localized photothermal effects arising from intense plasmonic coupling between adjacent Au NRs, which promote partial structural disassembly of the vesicles.

**Figure S31.** TEM images of Au NrCs in water after 5 min irradiation with 810 nm NIR light at different power intensities. All scale bars are 200 nm.

**Figure S32.** Stability of gold nanorods (Au NRs) under continuous 810 nm NIR LED irradiation. Au NRs with a longitudinal LSPR peak at 772 nm were continuously irradiated with an 810 nm NIR LED (120 mW cm^-2^) for 2 h. (a) UV-Vis-NIR extinction spectra recorded during continuous irradiation, showing no significant spectral changes. Representative TEM images of the Au NRs after (b) 0 min, (c) 5 min, and (d) 120 min of irradiation, showing no detectable morphological changes. Scale bars: 50 nm.

**Figure S33.** (a) Normalized extinction spectra of DNA-loaded Au NrCs before (purple) and after (red) NIR irradiation, normalized at the L-LSPR peak. (b) DLS size distribution profiles of DNA-loaded Au NrCs before (purple) and after (red) NIR irradiation.

**Figure S34.** Control experiments using Au NsCs exhibiting negligible extinction at 810 nm under NIR irradiation. (a) Normalized UV-Vis-NIR extinction spectra of Au NSs before (gray) and after (red) self-assembly into Au NsCs. (b) Normalized UV-Vis-NIR extinction spectra of Au NsCs before (red) and after (blue) 810 nm NIR irradiation for 30 min. (c) DLS volume distributions of Au NsCs before (red) and after (blue) NIR irradiation. (d) Representative TEM images of Au NsCs (i) before and (ii) after 30 min of NIR irradiation. (e) Temperature profiles of the Au NsC dispersion and a water control during 810 nm NIR irradiation (120 mW cm^-2^) for 20 min. (f) Time-dependent release of encapsulated FAM-DNA from Au NsCs during 810 nm NIR irradiation (120 mW cm^-2^) for 30 min. Data are presented as mean ± SD (*n* = 3). The negligible structural change and DNA release observed for the Au NsCs indicate that efficient longitudinal LSPR excitation of Au NRs is required for NIR-triggered capsule disassembly and cargo release.

**Figure S35.** Time-dependent fluorescence intensity of FAM-DNA in the supernatant of Au NrCs over 24 h without NIR irradiation. Fluorescence intensity at 520 nm is expressed as RFU. Data are presented as mean ± SD (*n* = 3).
